# Matricellular Protein Periostin Promotes Pericyte Migration in Fibrotic Airways

**DOI:** 10.3389/falgy.2021.786034

**Published:** 2021-12-03

**Authors:** Rebecca E. Bignold, Jill R. Johnson

**Affiliations:** School of Biosciences, College of Health and Life Sciences, Aston University, Birmingham, United Kingdom

**Keywords:** asthma, remodeling, pericyte, periostin, IL-13, TGF-β1

## Abstract

**Introduction:** Periostin is a matricellular protein that is currently used as a biomarker for asthma. However, its contribution to tissue remodeling in allergic asthma is currently unknown. We have previously demonstrated that tissue-resident mesenchymal stem cells known as pericytes are a key cell type involved in airway remodeling. This is thought to be caused the uncoupling of pericytes from the microvasculature supporting the large airways, facilitated by inflammatory growth factors and cytokines. It is hypothesized that periostin may be produced by profibrotic pericytes and contribute to the remodeling observed in allergic asthma.

**Methods:** Lung sections from mice with allergic airway disease driven by exposure to house dust mite (HDM) were stained using an anti-periostin antibody to explore its involvement in fibrotic lung disease. Human pericytes were cultured *in vitro* and stained for periostin to assess periostin expression. Migration assays were performed using human pericytes that were pretreated with TGF-β or periostin. ELISAs were also carried out to assess periostin expression levels in bronchoalveolar lavage fluid as well as the induction of periostin production by IL-13.

**Results:** Immunostaining indicated that pericytes robustly express periostin, with increased expression following treatment with TGF-β. Migration assays demonstrated that pericytes treated with periostin were more migratory. Periostin production was also increased in HDM exposed mice as well as in cultured pericytes treated with IL-13.

**Conclusion:** Periostin is produced by pericytes in response to TGF-β or IL-13, and periostin plays a key role in inducing pericyte migration. The increase in periostin expression in TGF-β or IL-13 treated pericytes suggests that IL-13 may trigger periostin production in pericytes whilst TGF-β modulates periostin expression to promote pericyte migration in the context of tissue fibrosis.

## Introduction

Allergic asthma is a common, yet complex condition involving the constriction of airways, airway wall thickening and overall reduction in airflow and increased airway hyperresponsiveness, driven by a Type 2 immune response to inhaled allergens such as house dust mite, pet dander, and pollen (1). The pathophysiology of asthma can be categorized by three aspects of airway remodeling: mucus hyperplasia, airway smooth muscle alterations, and deposition of extracellular matrix (ECM) proteins. The causes of remodeling are a much contested topic, but the general consensus is that myofibroblasts are responsible for the accumulation of contractile elements and ECM proteins in the airway wall (2–4). Pericytes are mesenchymal progenitor cells associated with the tissue microvasculature, and have been shown in organ fibrosis of varying etiology to be a primary source of myofibroblasts (5–10). In healthy tissue, pericytes maintain the structure and function of blood vessels and help control the transport of cells and molecules in and out of the vasculature (11). However, during inflammation-driven fibrosis, as observed in allergic asthma, pericytes have been observed to uncouple from capillaries within the airway wall and accumulate within and around airway smooth muscle bundles, with elevated expression of the myofibroblast marker α-smooth muscle actin (α-SMA) and a demonstrable contribution to airway hyperactivity in an allergen-driven model of allergic asthma (5). Additionally, pericytes are also thought be the prime source of ECM proteins in the lung and are major contributors to pulmonary fibrosis (12).

The complex interplay between inflammatory mediators that results in the uncoupling and migration of pericytes is an important area of investigation. By identifying the main driving force in this process, pericyte migration could be inhibited or even prevented, leading to improved lung function in allergic asthma. One inflammatory mediator that deserves attention is this regard is periostin, which is an extracellular matrix-derived bioactive peptide (matrikine) that has recently been implicated in Type 2-mediated tissue fibrosis (13, 14). Periostin is a matricellular protein that was first identified as a cell adhesion protein in 1993 (15). However, it is widely now associated with fibrosis and other pro-fibrotic cytokines. It has strong links with allergic asthma, with high serum levels of periostin being used as a biomarker of asthma severity (16). It has also been strongly linked to TGF-β, one of the most infamous pro-fibrotic cytokines, as a co-regulatory relationship has been suggested between these two mediators (17). This study aimed to identify the factors that stimulate periostin expression by pericytes and elucidate the contribution of periostin to airway remodeling in allergic asthma by assessing the expression of periostin in an allergen-driven mouse model of allergic airway disease (18, 19) and in cultured human pericytes.

## Methods

### *In vivo* House Dust Mite Model

Thirty female C57Bl/6 mice (6–8 weeks old) were purchased from Charles River and housed at the Aston University central animal facility under specific pathogen-free conditions. The mice were provided with food and water and exposed to a 12-h light-dark cycle. All mice were handled in compliance with UK Home Office regulations on animal care and welfare (Animals (Scientific Procedures) Act 1986). Allergic airway disease was induced using a previously described protocol (19). In brief, on 5 days per week over the course of five consecutive weeks, mice (*n* = 15) were anesthetized with isoflurane (Sigma-Aldrich, Missouri, USA) before being challenged with house dust mite (HDM) allergen. HDM extract (Citeq, The Netherlands) was suspended in sterile phosphate buffered saline (PBS; Sigma-Aldrich) at a final concentration of 2.5 mg/ml. Ten microliters of the solution was administered intranasally; control mice (*n* = 15) received 10 μl of sterile PBS using the same protocol. All animal experiments were carried out and recorded according to the ARRIVE guidelines.

### Scratch Assay

HPLPCs (human placental pericytes) from Promocell (Heidelberg, Germany) were cultured until between passage 5–10 in pericyte-specific medium (Promocell) with 1% antibiotic/antimycotic (ThermoFisher, Massachusetts, USA). All cell culture was performed in well plates or flasks coated with 2% gelatin powder (Sigma-Aldrich) in autoclaved distilled water. Once a monolayer of pericytes was formed they were treated with either 10 ng/ml TGF-β (Biolegend, California, USA), 100 ng/ml IL-13 (Biolegend), 100 ng/ml periostin (Biolegend) or 1 mM cinnamaldehyde (Sigma-Aldrich) for 48 h. A p200 pipette tip was used to scratch the monolayer and form a wound once in each well. The cells were then washed with PBS and transferred to serum-free low-glucose Dulbecco's Modified Eagle's Medium (DMEM; Sigma-Aldrich) with 1% antibiotic/antimycotic (Sigma-Aldrich) in order to arrest cell proliferation. Images of each scratch were taken at 100x before and after 24 h of incubation at 37°C and 5% CO_2_. The area of each scratch was calculated with ImageJ and the difference between the areas before and after the incubation were used to calculate the distance that was migrated.

### Immunostaining

Immunostaining was performed on cultured human pericytes (HPLPC; Promocell) and the large airways (tracheobronchial wholemounts) and lung tissue from mice.

HPLPC were cultured as previously described on coverslips coated in 2% gelatin. Cells were treated with 10 ng/ml TGF-β or 100 ng/ml periostin for 5 days before having the medium removed and being fixed with cold 100% ethanol for 10 min. Cells were then washed with PBS and blocked with 5% NGS (normal goat serum; Sigma-Aldrich) in 0.3% Triton-X-100/PBS for 60 min. The primary antibodies against periostin raised in rabbit (Abcam, Cambridge, UK; 1:200) and α-SMA raised in mouse (directly conjugated with Cy3; Sigma-Aldrich; 1:1000) were then added and incubated at room temperature for 60 min before being removed and washed again with PBS. The secondary antibody, AlexaFluor488 goat anti-rabbit (Invitrogen, Gillingham, UK; 1:500), was then added and incubated for 60 min. All antibodies were diluted in 0.3% Triton-X-100/PBS. Following this, the antibodies were removed and the coverslips were washed with PBS before being mounted with Fluoroshield (Sigma-Aldrich) mounting medium containing DAPI.

Whole lungs and tracheas were taken from the mice and stored in sucrose solution to cryopreserve the sample. Sucrose was rinsed off using PBS. The large airways (trachea and bronchi prepared and stained as a wholemount) were cleaned of extraneous tissue and pinned down onto Sylgard-coated plates (Sigma-Aldrich). Whole lungs were embedded in TissueTek OCT (Sakura Finetech, Newbury, UK) and frozen at −80°C. Sections (10 μm) were then cut using a cryostat (Leica, Germany) and mounted on Superfrost Plus slides (Fisher Scientific, Loughborough, UK). The slides were stored at −80°C. Prior to staining, slides were warmed to room temperature. A hydrophobic marker was used to outline the tissue sections before they were blocked with 5% NGS in 0.3% Triton-X-100/PBS for 2 h. After this, the blocking solution was washed off with PBS and the slides were incubated overnight at room temperature with primary antibodies against periostin raised in rabbit (Abcam), α-SMA raised in mouse (Abcam), or CD31 raised in Armenian hamster (Biolegend). Following incubation, the primary antibody was washed off with PBS containing 0.3% Triton-X, and the secondary antibody (AlexaFluor488 goat anti-rabbit (Invitrogen), AlexaFluor555 goat anti-mouse (Invitrogen), or AlexaFluor649 goat anti-hamster (Invitrogen) was added and incubated again for 2 h. Slides were then washed again with PBS and mounted using Fluoroshield with DAPI (Sigma-Aldrich). Lung sections and tracheobronchial wholemounts were imaged at the ARCHA Advanced Imaging Facility at Aston University, employing a SP5 TCS II MP confocal microscope (tracheobronchial wholemounts; Leica), an EVOS XL microscope (ThermoFisher), or a widefield fluorescent microscope (Leica).

### ELISA

Anti-periostin ELISAs (R&D Systems, Minnesota, US) were performed on supernatant harvested from cultured HPLPCs treated with 10 ng/ml TGF-β (Biolegend), 10 ng/ml EGF (Biolegend), 10 ng/ml VEGF (Biolegend), 100 ng/ml IL-13 (Biolegend), 100 ng/ml periostin (Biolegend) or 0–2.5 mM cinnamaldehyde (Sigma-Aldrich) and on bronchoalveolar lavage fluid harvested from HDM treated (and control) mice prepared as previously described. All ELISAs were performed as per the manufacturer's instructions.

### Statistical Analysis

All results are shown as mean ± standard deviation. GraphPad Prism 8 was used for data processing and statistical analyses. Differences were evaluated by Student's *t*-test for two groups, by one-way analysis of variance (ANOVA) for multiple groups with the Tukey *post-hoc* test, or by two-way ANOVA with Šídák's multiple comparisons test as appropriate. Differences were deemed to be statistically significant when a *p*-value <0.05 was obtained (*p < 0.05, **p < 0.01, ***p < 0.001).

## Results

### Periostin Expression Is Increased in Pericytes Following Chronic Aeroallergen Exposure in Mice

**Figure 1:**
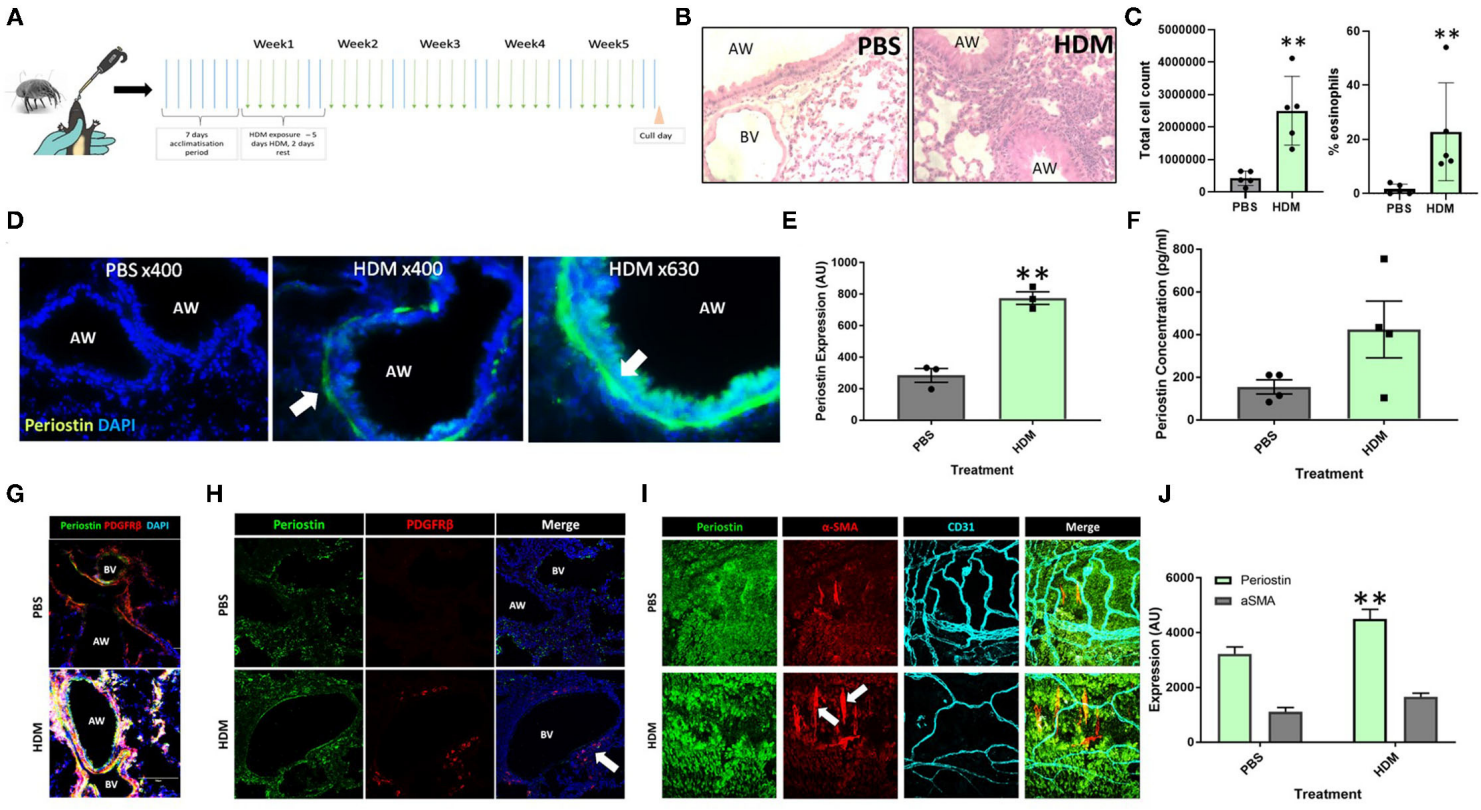
Periostin expression is increased following chronic aeroallergen exposure in mice. **(A)** Schematic diagram of HDM-induced allergic airway inflammation in mice. **(B)** Female C57/Bl6 mice (6–8 weeks old) were exposed to either sterile PBS (10 μl intranasally) or house dust mite extract (HDM; 25 μg in 10 μL) 5 days a week for five consecutive weeks. Hematoxylin and eosin stained lung sections from PBS and HDM-exposed mice. **(C)** Bronchoalveolar lavage fluid total cell counts and percentage of eosinophils. **(D)** At the end of the allergen exposure protocol, lung sections obtained from PBS control and HDM-exposed mice were stained with an anti-periostin antibody (green) and DAPI to stain nuclei (blue). Images were taken at 400x and 630x magnification as stated. Arrows indicate periostin positive cells. **(E)** Expression of periostin was calculated in the area of interest around each airway using ImageJ. **p < 0.01, *n* = 3 representative of two independent experiments. **(F)** Bronchoalveolar lavage samples were collected and the periostin content was assessed by ELISA. *n* = 4 representative of two independent experiments. **(G,H)** Lung sections were stained for periostin (green), the pericyte marker PDGFRβ (red), and cell nuclei (DAPI, blue) to demonstrate the presence of periostin-expressing pericytes around the airways and blood vessels of HDM-exposed mice. **(I)** Tracheobronchial whole mounts were stained for the mesenchymal cell marker α-smooth muscle actin (α-SMA; red), the endothelial cell marker CD31 (cyan), and periostin (green) and imaged at 400x magnification; arrow indicate periostin-positive pericytes. **(J)** The expression of periostin and α-SMA in tracheobronchial whole mounts was calculated using ImageJ. AW, airway; BV, blood vessel. **p < 0.01, *n* = 3–5 representative of two independent experiments.

Mice that had been subjected to 5 weeks of aeroallergen exposure (Figure 1A) demonstrated robust peribronchial inflammation with a high percentage of infiltrating eosinophils (Figures 1B,C), indicating the establishment of a robust Th2 inflammatory response to HDM exposure. The lung tissue was found to express increased levels of periostin expression, particularly in remodeled large airways, as compared to healthy control lungs. As shown in Figure 1D (white arrow), robust periostin expression was observed in the subepithelial region of the large airways (bronchi and bronchioles). Conversely, the lungs of PBS-exposed mice showed a very low level of periostin expression. This was quantified through image analysis in ImageJ. where a region of interest was selected at a consistent distance around the airways and the density of positive periostin staining was calculated. This analysis showed that the large airways of mice that were exposed to HDM had significantly (*p* < 0.05) higher expression of periostin the airways of healthy mice (Figure 1E). The concentration of soluble periostin was measured by ELISA in the bronchoalveolar lavage fluid of mice exposed to HDM and control mice (Figure 1F), with a trend toward higher periostin levels in mice exposed to HDM.

Further assessments of lung tissue stained for periostin expression demonstrated that periostin was primarily expressed by airway epithelial cells and pulmonary pericytes (Figures 1D,G), with particularly robust expression observed in pericytes that had uncoupled from the supporting microvasculature (white arrows in Figures 1H,I). Similar to what was observed in the subepithelial region of the large airways in lung sections (Figure 1E), periostin expression was significantly increased in the tracheobronchial whole mounts (Figure 1J).

### Type 2 Inflammatory Mediators Stimulate Periostin Expression by Pericytes

**Figure 2:**
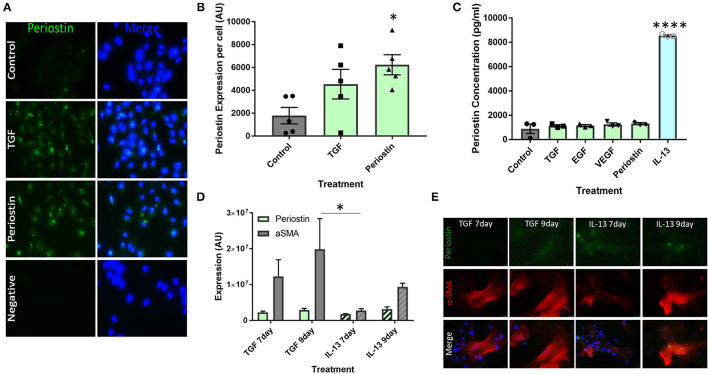
Type 2 inflammatory mediators stimulate periostin expression by pericytes. **(A,B)** Immunostaining performed on pericytes grown in pericyte medium and treated with 10 ng/ml of TGF-β or 100 ng/ml of periostin for 24 h. Cells were stained with an anti-periostin antibody (green) and the nuclear stain DAPI (blue). Images were taken at 400x magnification and intensity of periostin stain was calculated with ImageJ. Intensity of stain per field of view was divided by the number of cells in order to determine periostin expression per cell. *p < 0.05, *n* = 5 representative of two independent experiments. **(C)** Cultured pericytes were treated with 10 ng/ml TGF-β, 10 ng/ml EGF, 10 ng/ml VEGF, 100 ng/ml IL-13 or 100 ng/ml Periostin in pericyte medium for 7 days before the supernatant was harvested. The periostin content was assessed using an anti-periostin ELISA kit. ****p < 0.0001, *n* = 3 representative of two independent experiments. **(D,E)** Cultured pericytes were treated with 10 ng/ml TGF-β or 100 ng/ml IL-13 for either 7 or 9 days. Cells were stained with an anti-periostin antibody and an anti-αSMA antibody. The images were taken at 400x magnification and quantifications were made using ImageJ. *p < 0.05, *n* = 3–4 representative of two independent experiments.

Cultured pericytes were treated with the pro-fibrotic mediators TGF-β and periostin in order to simulate the conditions of fibrosis seen in allergic asthma. Untreated pericytes were found to express low levels of periostin under control conditions, as seen in Figure 2A. This production was increased following treatment with TGF-β and periostin (Figure 2A) and quantified using ImageJ (Figure 2B). Periostin treatment significantly increased periostin production (*p* < 0.05), to a greater extent than TGF-β treatment.

ELISAs were utilized to measure soluble periostin protein secreted by pericytes treated with a number of profibrotic mediators, selected to explore the relationship between these inflammatory mediators, periostin expression, and fibrosis. Most mediators did not induce the production of soluble periostin in cultured pericytes, i.e., TGF-β, EGF, VEGF and periostin (Figure 2C). Conversely, the supernatants from pericytes treated with IL-13 contained a significantly higher concentration of periostin (*p* < 0.0001), with an almost seven-fold increase.

Cultured pericytes were then treated with TGF-β or IL-13 and assessed after 7 days or 9 days of exposure. Cells were stained for periostin expression as well as the pericyte/mesenchymal cell marker α-SMA (Figures 2D,E). In both treatments, periostin and α-SMA expression were higher following 9 days of exposure to both TGF-β and IL-13 compared to 7 days. Periostin expression was similar following TGF-β and IL-13 treatment; however, TGF-β treatment induced a much higher expression of α-SMA than IL-13 treatment.

### Type 2 Inflammatory Mediators Stimulate Pericyte Migration

**Figure 3:**
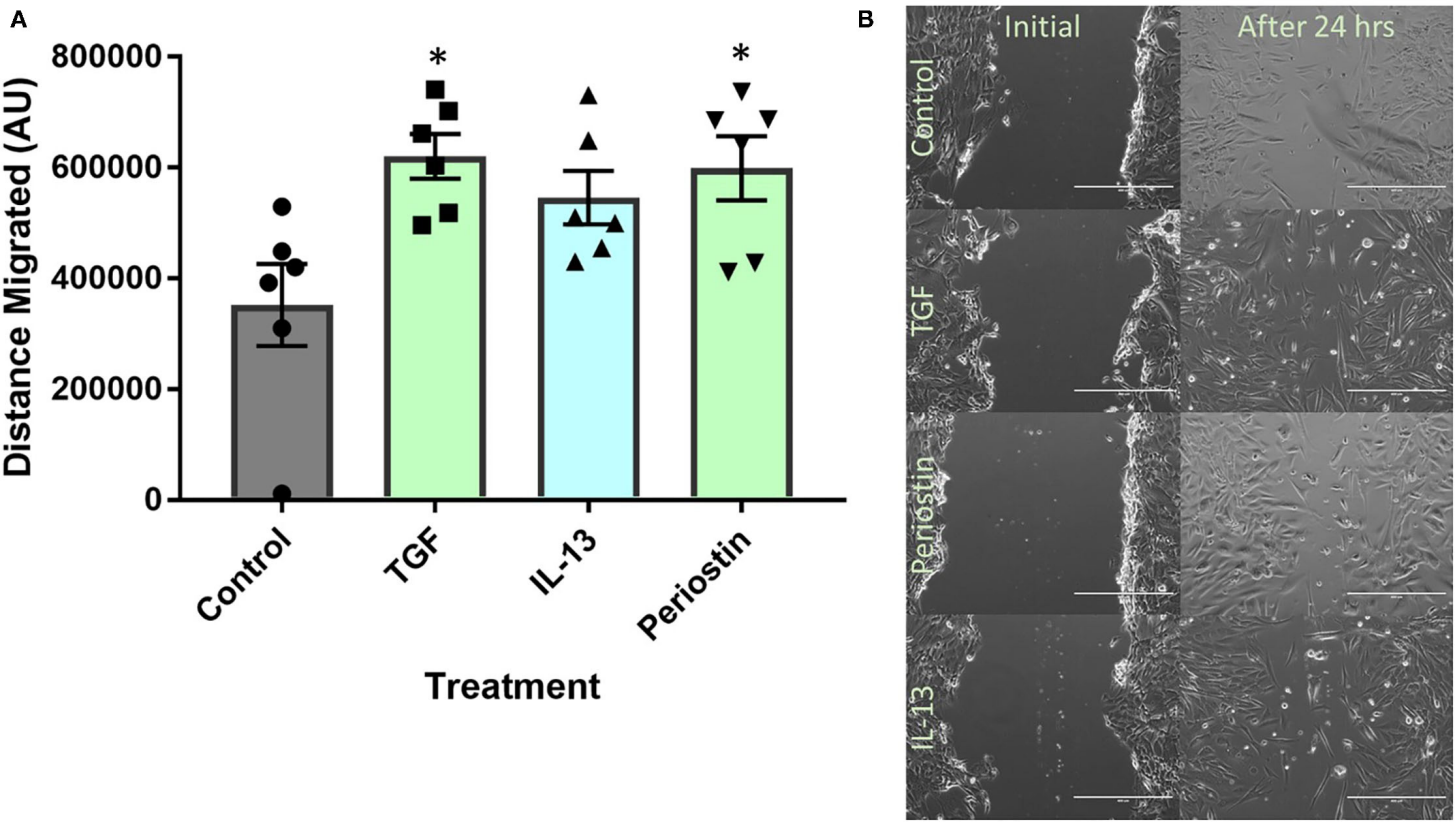
Type 2 inflammatory mediators stimulate pericyte migration. **(A)** Scratch assay performed on pericytes that were grown in pericyte medium and treated with 10 ng/ml of TGF-β, 100 ng/ml of IL-13 or 100 ng/ml of periostin for 48 h. Cells were transferred into serum-free medium and a scratch was made in each monolayer using a p200 pipette tip. **(B)** Images were taken at 100x magnification immediately after scratching and 24 h later. The scratch width was determined using ImageJ and the average distance of cell migration was calculated. *n* = 6 representative of two independent experiments, *p < 0.05 vs. untreated cells.

Figure 3A shows that treatment with TGF-β and periostin significantly increased the migration of pericytes over 24 h. The images in Figure 3B are representative of each condition and also show a decrease in the scratch width over 24 h. In these images, the cells treated with TGF-β, periostin and IL-13 had a smaller remaining scratch area than the untreated cells. As the pericytes were incubated in serum-free medium, cell proliferation was arrested and therefore any increase in cell mass in the wound was likely from pericytes that had moved into the scratch area.

### Cinnamaldehyde Treatment Suppresses Periostin Expression and Pericyte Migration

**Figure 4:**
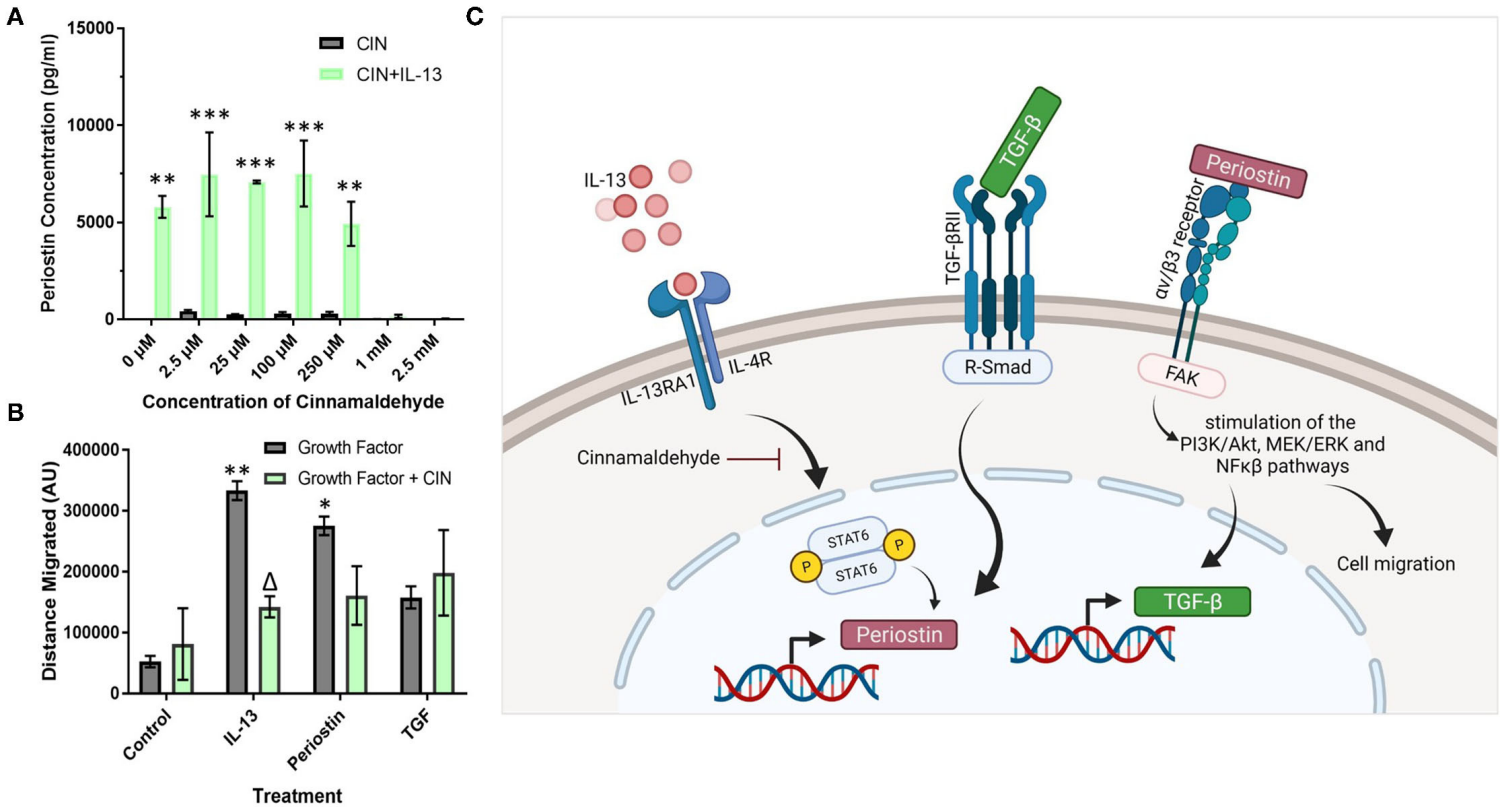
Cinnamaldehyde treatment suppresses periostin expression and pericyte migration. **(A)** Cultured pericytes were treated with 100 ng/ml IL-13 in pericyte media for 7 days with the addition of cinnamaldehyde for the last 3 days at the concentration stated in **(A)** or only cinnamaldehyde at the stated concentration for 3 days. The supernatants were harvested and the periostin content was assessed by ELISA. **p < 0.01, ***p < 0.001 vs. the same cinnamaldehyde concentration without IL-13, *n* = 2 representative of two independent experiments. **(B)** Scratch assay performed on pericytes that were grown in pericyte medium and treated with 10 ng/ml of TGF-β, 100 ng/ml of IL-13 or 100 ng/ml of periostin for 7 days with 1 mM cinnamaldehyde added for the last 3 days. Cells were transferred into media lacking serum and a scratch was made in each monolayer using a p200 pipette tip. Images were taken at 100x magnification immediately after scratching and 24 h later. The scratch width was determined using ImageJ and the average distance of cell migration was calculated. *n* = 3 representative of two independent experiments, Δ = *p* < 0.05 vs. the same growth factor treatment without cinnamaldehyde, *p < 0.05, **p < 0.01 vs. control treatment without cinnamaldehyde. **(C)** Schematic diagram of the putative signaling pathways regulating periostin expression in pericytes (created using Biorender).

Cinnamaldehyde (CIN) was explored as an IL-13 inhibitor to reduce the effects of periostin, particularly on pericyte migration. First, the effects of CIN on periostin production by IL-13 treated pericytes were observed using ELISA to elucidate the optimum concentration of CIN to use in further studies (Figure 4A). Then, this concentration was used to treat cultured pericytes alongside IL-13, periostin, and TGF-β to determine whether CIN can prevent the migration of pericytes, assessed using the scratch assay.

Between the concentrations 0–250 μM, CIN had little effect on the production of periostin in cells treated with IL-13 as the concentration of periostin was maintained at around 7000 pg/ml, similar to the pericytes that had been treated solely with IL-13 (Figure 4A). However, at higher concentrations of CIN, i.e., 1 mM and 2.5 mM, the concentration of periostin dropped below the detection limit of the ELISA, suggesting that the IL-13 had been successfully inhibited. Without IL-13, the pericytes failed to produce any detectable levels of periostin at any concentration of CIN.

As the lowest concentration of CIN to produce an inhibitory effect was 1 mM, this concentration was used in the migration assay (Figure 4B). Treating pericytes with IL-13 and periostin significantly increased the migration of pericytes (*p* < 0.01), as previously shown in Figure 3. The addition of CIN to pericytes treated with both IL-13 and periostin decreased the migration of pericytes, with CIN significantly reducing the migration of IL-13 treated pericytes (*p* < 0.05). There was no difference to the migration of pericytes treated with TGF-β with or without CIN; similarly, CIN treatment alone had no effect on the migration ability of untreated pericytes.

## Discussion

Periostin has been shown to contribute to several aspects of tissue remodeling in allergic asthma (13, 16). Imaging periostin *in situ* in fibrotic lung tissue indicated a layer of cells in the subepithelial space of the airway that expressed periostin very strongly. As these periostin expressing cells were not present in the lung sections from healthy, PBS exposed mice, periostin evidently contributes to the tissue remodeling seen around the large airways in allergic asthma (5, 19). Previous studies have shown that pericytes uncouple from the microvasculature supporting the large airways and migrate into the smooth muscle layer of the airway wall where they contribute to airway wall remodeling and airway hyperresponsiveness following differentiation into myofibroblasts (5, 20). In the present study, these uncoupling PDGFRβ-positive pericytes in the airways of HDM-exposed mice were found to highly express periostin, demonstrated using both tracheobronchial whole mounts and lung sections (Figures 1H,I), supporting the notion at periostin expression by pericytes is associated with increased migratory capacity.

Periostin was also shown to be present in the bronchoalveolar lavage fluid from mice exposed to HDM (Figure 1C). This indicates that there was a large concentration of periostin present in the inflamed lung, which may contribute to the remodeling observed in allergic asthma. However, as this lavage was collected from the airway lumen, it may not accurately depict the levels of periostin within the subepithelial space where it would interact with cells such as pericytes during the induction of airway wall remodeling. However, serum periostin has been investigated as a biomarker for eosinophilic inflammation and airway remodeling in allergic asthma (16), supporting the notion that elevated periostin levels in multiple body compartments is a feature of allergic airway inflammation and remodeling. The increase of periostin in both pericytes and epithelial cells in the trachea (Figures 1I,J) and overall lung tissue of the lungs of HDM-exposed mice justified further investigations into the impact periostin on pericyte migration and differentiation into myofibroblasts *in vitro*.

Cultured human pericytes were capable of producing high levels of periostin under pro-fibrotic conditions (Figures 2A,B). The production of periostin by pericytes is important as it shows that pericytes are an important driver of the profibrotic microenvironment and contribute to the cascade of mediators that initiate fibrosis. These results (Figures 2A,B) also suggest a co-regulatory relationship between TGF-β and periostin, as treatment with TGF-β also induced the production of periostin.

The data shown in Figure 2C confirms the suggestion that IL-13 is the initiating factor of periostin production. This has been described in several different cell types, such as epithelial cells and fibroblasts, but has not yet been linked to pericytes (13, 21). Treatment with any other of the tested cytokines did not lead to the release of periostin by pericytes. We had already demonstrated that TGF-β treatment increased the expression of periostin in pericytes (Figures 2A,B), but elevated intracellular expression of periostin did not translate into periostin release from pericytes into the supernatant under these conditions. Periostin has been observed in both the cytoplasm and the extracellular matrix, which may indicate that it plays several different roles depending on its location (22–24). Further studies should be completed to explore the localization of periostin in fibrotic conditions and the resulting effects on myofibroblast migration, differentiation, and collagen deposition.

The contribution of periostin to enhanced pericyte migration was shown in both Figures 3A,B, 4B. The migration of pericytes is a key process in the initiation of airway fibrosis in response to allergen exposure, as pericytes are required to uncouple from the vasculature and migrate toward the airways in order to contribute to the airway thickening and differentiation into myofibroblasts (5). Studies in other organs, primarily in the context of glioblastoma, have shown increased periostin expression along with increased migratory capacity in pericytes, as a mechanism driving angiogenesis during tumor growth (25). By highlighting periostin as a possible contributing factor to cell migration, this process may be targeted and arrested early on. With both TGF-β and periostin having a similar effect on pericyte migration in scratch assays, as shown in Figure 3, this suggests that there is a relationship between TGF-β and periostin as previously described by several other groups (26, 27).

Cinnamaldehyde is a compound with antioxidant properties, and has been shown to inhibit IL-13 activity (28). Thus, cinnamaldehyde treatment was explored as a possible means to reduce the amount of periostin present in the system and thereby suppress its effects on pericytes and tissue fibrosis. The impact of CIN was explored at different doses (Figure 4A) in order to determine the optimum dose to achieve the inhibition of periostin expression. As the concentration of IL-13-induced periostin production dropped significantly at 1 mM CIN, this was the dose selected for subsequent experiments. This concentration was then used to inhibit periostin-induced pericyte migration (Figure 4B). There was a significant change in the migratory behavior of IL-13-treated pericytes when CIN was added, suggesting that CIN successfully blocked the activity of IL-13 and its downstream effects on periostin expression and pericyte migration. IL-13 treated pericytes also migrated most effectively, which confirms that the sharp increase in periostin production induces by IL-13 contributes to migration. Periostin-treated pericytes also migrated effectively, although CIN treatment only reduced this slightly, likely due to its inhibitory step being further upstream. TGF-β treatment did not have a considerable effect on pericyte migration, and this was not impacted by CIN treatment. This may be due to the fact that TGF-β also stimulates pericyte differentiation into myofibroblasts, as previously demonstrated in pericytes derived from the kidney, i.e., mesangial cells (29). Thus, our experiments suggest that IL-13 and TGF-β cooperatively increase periostin expression and secretion by pericytes. After secretion, periostin subsequently enhances pericyte migratory capacity and TGF-β expression, likely *via* integrin αvβ3, representing a critical positive feedback loop in the induction of airway remodeling in response to allergic inflammation. The putative signaling pathways involved in increased pericyte migration in response to IL-13, TGF-β, and periostin are shown in Figure 4C. Studies are underway to explore this further in pulmonary pericytes in the context of allergic airway inflammation.

This study has several important limitations. First the causality of elevated periostin expression as a driver of increased pericyte migration has not been conclusively demonstrated. Experiments employing periostin knockout/knockdown strategies should be performed to explore this hypothesis. Second, the reversibility of periostin overexpression in the lungs of mice exposed to allergen has not yet been explored, which may be informative when considering targeting periostin expression therapeutically. Finally, the *in vitro* experiments were performed on placental pericytes (which are commercially available) rather than human pulmonary pericytes, owing to the difficulty of obtaining fresh human lung specimens during the COVID-19 pandemic. Future work in this field should explore the similarities and differences in pericytes from different organs.

## Conclusion

Periostin is an often overlooked, yet important mediator in fibrosis and, in particular, allergic asthma. Pericytes have been shown to actively produce periostin in a profibrotic environment both *in vitro* and *in vivo*. This study has demonstrated that the production of periostin is initiated by IL-13, a Type 2 cytokine with well-known roles in allergic asthma. Elevated periostin levels were found to promote the migration of pericytes, a key event in airway wall remodeling in allergic airway disease. This increased migration was prevented by the addition of cinnamaldehyde, an IL-13 inhibitor that shows promise as a future pharmaceutical intervention. Further studies should be completed in order to test the full capabilities of cinnamaldehyde as well as to elucidate the full effects of IL-13 and periostin in the fibrotic lung.

## Data Availability Statement

The raw data supporting the conclusions of this article will be made available by the authors, without undue reservation.

## Ethics Statement

The animal study was reviewed and approved by the Aston University Bioethics Committee.

## Author Contributions

RB and JJ conceived of the study. RB carried out the experiments and contributed to data analysis and writing the manuscript. JJ contributed to writing the manuscript and supervised the study. All authors contributed to the article and approved the submitted version.

## Funding

The authors gratefully acknowledge financial support from the School of Biosciences at Aston University for the Ph.D. scholarship to RB and to the UK Medical Research Council for the New Investigator Research Grant to JJ (MR/K011375/1).

## Conflict of Interest

The authors declare that the research was conducted in the absence of any commercial or financial relationships that could be construed as a potential conflict of interest.

## Publisher's Note

All claims expressed in this article are solely those of the authors and do not necessarily represent those of their affiliated organizations, or those of the publisher, the editors and the reviewers. Any product that may be evaluated in this article, or claim that may be made by its manufacturer, is not guaranteed or endorsed by the publisher.
